# Synthesis of lysine methyltransferase inhibitors

**DOI:** 10.3389/fchem.2015.00044

**Published:** 2015-07-23

**Authors:** Chunngai Hui, Tao Ye

**Affiliations:** Department of Applied Biology & Chemical Technology, The Hong Kong Polytechnic UniversityHung Hom, Hong Kong

**Keywords:** epigenetics, lysine methyltransferase, inhibitors, synthesis, S-adenosyl-l-methionine

## Abstract

Lysine methyltransferase which catalyze methylation of histone and non-histone proteins, play a crucial role in diverse biological processes and has emerged as a promising target for the development of various human diseases, including cancer, inflammation, and psychiatric disorders. However, inhibiting lysine methyltransferases selectively has presented many challenges to medicinal chemists. During the past decade, lysine methyltransferase inhibitors covering many different structural classes have been designed and developed. In this review, we describe the development of selective, small-molecule inhibitors of lysine methyltransferases with an emphasis on their discovery and chemical synthesis. We highlight the current state of lysine methyltransferase inhibitors and discuss future directions and opportunities for lysine methyltransferase inhibitor discovery.

## Introduction

**Figure 1:**
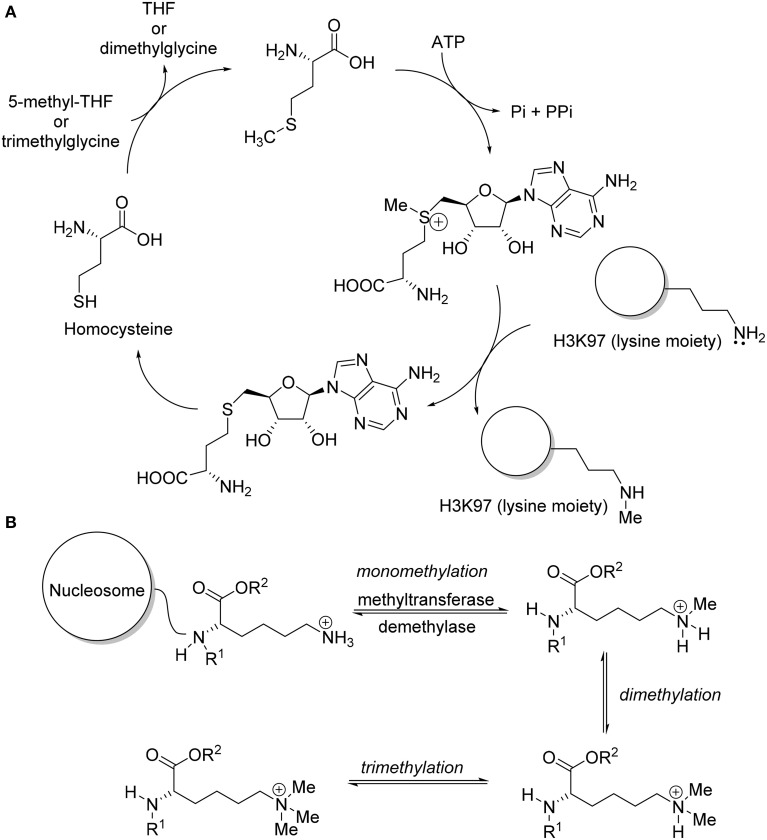
**(A)** Mechanism of DOT1L in methylation of lysine moiety of protein H3K79. **(B)** Specific lysine methyl transferase methylated lysine residues in histone lysine tail.

Epigenetic aberrations often lead to cancer and other human diseases. Recent basic studies on histone methyltransferases indicated that deregulation of histone methylation plays a critical role in human carcinogenesis (Copeland et al., 2009; Spannhoff et al., 2009; Chi et al., 2010; Arrowsmith et al., 2012; Helin and Dhanak, 2013). In fact, protein lysine methylation has attracted considerable attention since the discovery of the first histone lysine methyltransferase, Suv39H1, in 2000 (Rea et al., 2000). After the discovery of Suv39H1 with lysine methyltransferases activities, additional proteins with methyltransferase activity were reported, such as G9a/GLP (Tachibana et al., 2002, 2005), EZH2 (Cao et al., 2002), MLLs (Milne et al., 2002), SET2 (Strahl et al., 2002), SET7/9 (Wang et al., 2001), DOT1 (Feng et al., 2002; Van Leeuwen et al., 2002), and SETD8 (Nishioka et al., 2002). These methyltransferases are classified as either SET-domain containing or non-SET-domain containing enzymes for methylation of lysine residue on histone (Spannhoff et al., 2009). The function of these enzymes includes serving as catalysts for the transfer of methyl group(s) from the co-factor *S*-adenosyl-L-methionine (SAM) to the lysine residues of histone (Yao et al., 2011) (Figure 1A). Specific lysine methyltransferase catalyzed the methylation of lysine residue in a site-dependent manner and catalyze the formation of distinct methylation state (Wagner et al., 2014) (Figure 1B). New findings of somatic mutations and mis-expression of genes coding for histone methyltransferase enzymes provided a strong impetus for therapeutic intervention via pharmacological modulation. Epigenetic drug development, especially the discovery of small molecule inhibitors for histone methyltransferases, has quickly gained widespread interest (Pachaiyappan and Woster, 2014). Substantial review effort was made on bioactivities and biological mode of actions of the recently developed lysine methyltransferase inhibitor (Copeland et al., 2009; He et al., 2012; Helin and Dhanak, 2013; Itoh et al., 2013; Knapp and Weinmann, 2013; Tian et al., 2013; Wang and Patel, 2013; Bojang and Ramos, 2014; Dhanak and Jackson, 2014). So far, no review work on chemical synthesis of lysine methyltransferase inhibitors has been reported. The aim of this review is to provide a concise summary of the research published in the recent years, with an emphasis on the chemical syntheses of lysine methyltransferase inhibitors. Special attention will be paid to inhibitors with prominent biological activities targeting protein lysine methyltransferases such as G9a/GLP, Suv391H1, DOT1L, EZH2, and SETD8.

## Suv39H1

Jenuwein and co-workers discovered that human Suv39H1 protein possesses histone H3K9 methyltransferases activities. Analysis of the Suv39H1 protein revealed that it carried a conserved motif of 130 amino acids which is corresponding to the SET domain (Jenuwein et al., 1998). Later, it was found that the SET domain was responsible for the histone lysine methylation function (Rea et al., 2000). Greiner and co-worker discovered that the fungal metabolite chaetocin **1** (Hauser et al., 1970), exhibited selectivity toward Su(var)3-9 methyltransferase (IC_50_ = 0.6 μM) (Greiner et al., 2005) is competitive with reactive methyl donor *S*-adenosyl-methionine (SAM) to facilitate enzymatic inhibition upon kinetic study (Figure 1). More recent work revealed that Chaetocin **1** was a non-specific inhibitor because it can inhibit many other unrelated enzymes (Cherblanc et al., 2013a). It also showed inhibitory effect toward G9a methyltransferase (Iwasa et al., 2010; Cherblanc et al., 2013b).

**Figure 2:**
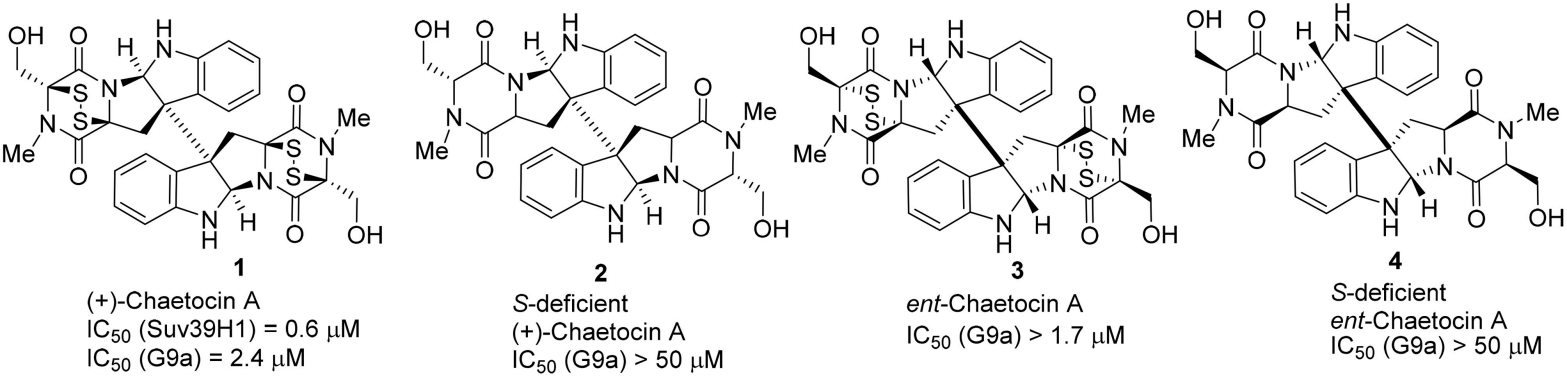
**Example of inhibitors targeting protein Suv39H1**.

Structurally, Chaetocin **1** is classified as a complex epidithiodiketopiperazine (ETP) alkaloid (Iwasa et al., 2011b). Its total synthesis was accomplished by Sodeoka (Iwasa et al., 2010) and Movassaghi (Kim and Movassaghi, 2010), respectively in 2010. Sodeoka and coworkers also reported the synthesis of *ent*-chaetocin A **3** and the corresponding sulfur-deficient analogs (Iwasa et al., 2010, 2011a; Sodeoka et al., 2012; Fujishiro et al., 2013). Biological studies revealed that chaetocin A **1** and its *ent*-isomer **3** (*ent*-chaetocin A) inhibited G9a methyltransferase in similar extent (IC_50_ = 2.4 and 1.7 μM, respectively) (Iwasa et al., 2010). However, sulfur-deficient analog of chaetocin A **2** and its *ent*-isomer **4** were inactive toward G9a. (IC_50_ > 50 μM) (Figure 2).

**Scheme 1:**
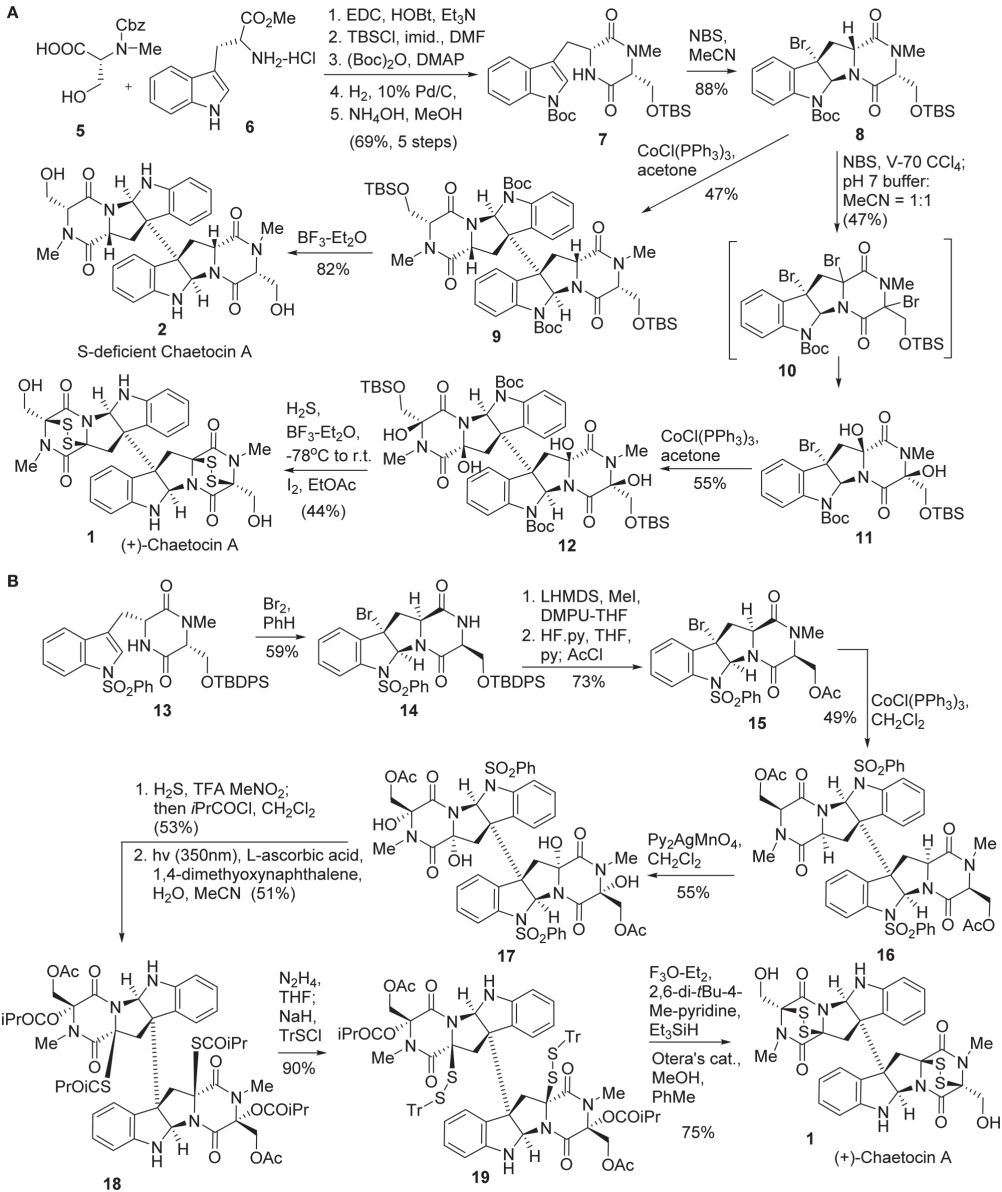
**(A)** Total synthesis of (+)-Chaetocin A and its *S*-deficient analog by Sodeoka et al. **(B)** Total synthesis of (+)-Chaetocin A by Movassaghi and co-workers.

Sodeoka and co-workers reported the first total synthesis of chaetocin A **1** in 2010 (Iwasa et al., 2010) (Scheme 1A). Diketopiperazine **7** was synthesized in five steps from Cbz protected N-methyl-D-serine **5** (Aurelio et al., 2003) and commercially available D-tryptophan methyl ester **6**. Bromocyclization of **7** was promoted by NBS to afford **8** in 88% yield without formation of other diastereomers (Movassaghi et al., 2008; Kim et al., 2009). Stereoselective bromination of **8** was carried out using 2,2′-azobis-(2,4-dimethyl-4-methoxyvaleronitrile) (V-70) (Kita et al., 1997) as a radical initiator to afford *tri*-brominated intermediate **10**, which was then treated with phosphate buffer to give rise to **11** in 47% yield. Reductive coupling of the freshly prepared diol **11** under Movassaghi Co(I)-catalyzed radical dimerization protocol (Movassaghi and Schmidt, 2007) furnished **12** in 55% yield. Construction of the disulfide bridge in **1** was accomplished by treating of dimmer **12** with H_2_S and BF_3_-Et_2_O, followed by I_2_ to afford chaetocin A **(1)** in 44% yield. Sodeoka also disclosed the method for the preparation of sulfur-deficient chaetocin A **2**. Direct reductive coupling of **8** using Cobalt(I) as a catalyst produced dimer **9** in 47% yield and successive desilylation afforded sulfur-deficient chaetocin A **2** in 82% yield. In addition, the enantiomer of chaetocin A **(3)** (*ent*-chaetocin A) and its sulfur-deficient analog **4** were also prepared by the use of the same procedure (Iwasa et al., 2010, 2011a).

Movassaghi and co-workers reported the second total synthesis of chaetocin **1** in 2010 (Kim and Movassaghi, 2010) (Scheme 1B). The synthesis began with bromocyclolization of diketopiperazine **13** to afford **14** in 59% yield with high diastereoselectivity (Movassaghi et al., 2008). Successive *N*-methylation using MeI/LiHMDS, desilylation, and acetoxylation afforded **15**, which was then subjected to Cobalt(I)-catalyzed reductive coupling (Movassaghi and Schmidt, 2007; Movassaghi et al., 2008) to afford dimeric product **16** in 49% yield. Stereoselective *tetra*-hydroxylation of **16** was carried out by the action of Py_2_AgMnO_4_ to give rise to **17** in 55% yield (Kim et al., 2009). The high chemo- and stereo-selectivity of the above transformations enabled the advanced intermediates to be prepared in gram scale. Exposure of tetraol **17** to TFA and H_2_S allowed the formation of a *bis*thioaminal intermediate with high stereoselectivity in which both the diol and dithiol were immediately protected as the corresponding ester or thioester. Subsequent *N*_1_-desulphonylation under irradiation with a Black-light phosphor-coated lamp, 1,4-dimethyoxynaphthalene as photosensitizer and ascorbic acid as terminal reductant furnished the desired product **18** in 51% yield (Hamada et al., 1986). Chemoselective hydrazinolysis of thioester **18** afforded disulfide **19** in 90% yield. Sequential ionization of triphenylemthyl (Tr) group and cyclization with the loss of triphenylcarbocation afforded the disulfide-bridge-carrying chaetocin A precursor in 82% yield. Subsequent hydrolysis of acetate using Otera's catalyst afforded chaetocin A **1**.

## G9a/GLP

G9a and GLP are the primary enzymes for mono and dimethylation at Lys 9 of histone H3 (H3K9me1 and H3K9me2), and exist predominantly as a G9a–GLP heteromeric complex that appears to be a functional H3K9 methyltransferase *in vivo* (Shinkai and Tachibana, 2011). G9a-GLP complex regulates a wide range of biological activities including germ cell development, meiosis, DNA replication, cell proliferation, and cancer cell formation. Overexpression of protein G9a is observed in many cancers, including prostate, lung, colon cancer, and lymphocytic leukemia (Chen et al., 2010; Shinkai and Tachibana, 2011). It was reported that overexpression of G9a led to increasing metathesis and invasion in lung cancer (Chen et al., 2010). Since G9a is overexpressed in many different types of cancers, and has been shown to be responsible for various aspects of tumorigenesis, including cellular differentiation, proliferation, and epithelial to mesenchymal, indicates that G9a could be a feasible target for cancer therapy.

**Figure 3:**
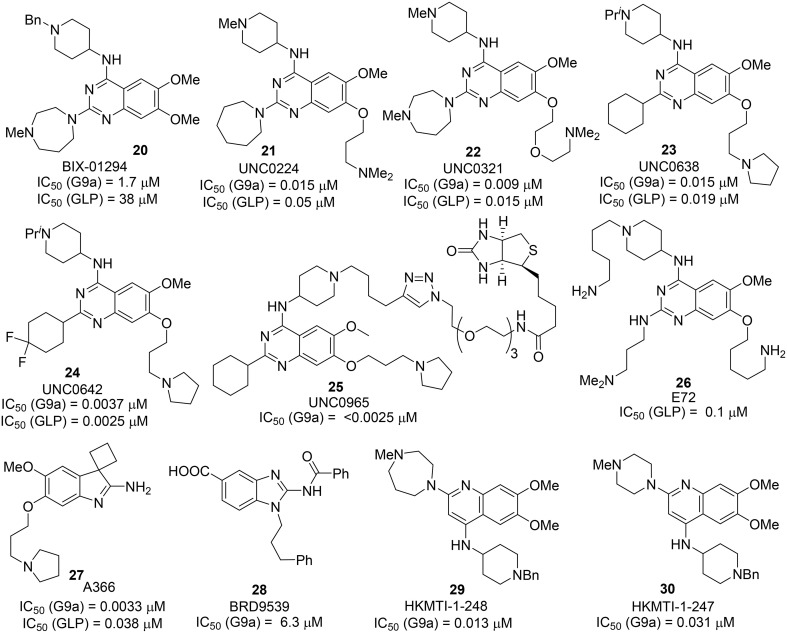
**Example of inhibitors targeting protein G9a-GLP**.

**Scheme 2:**
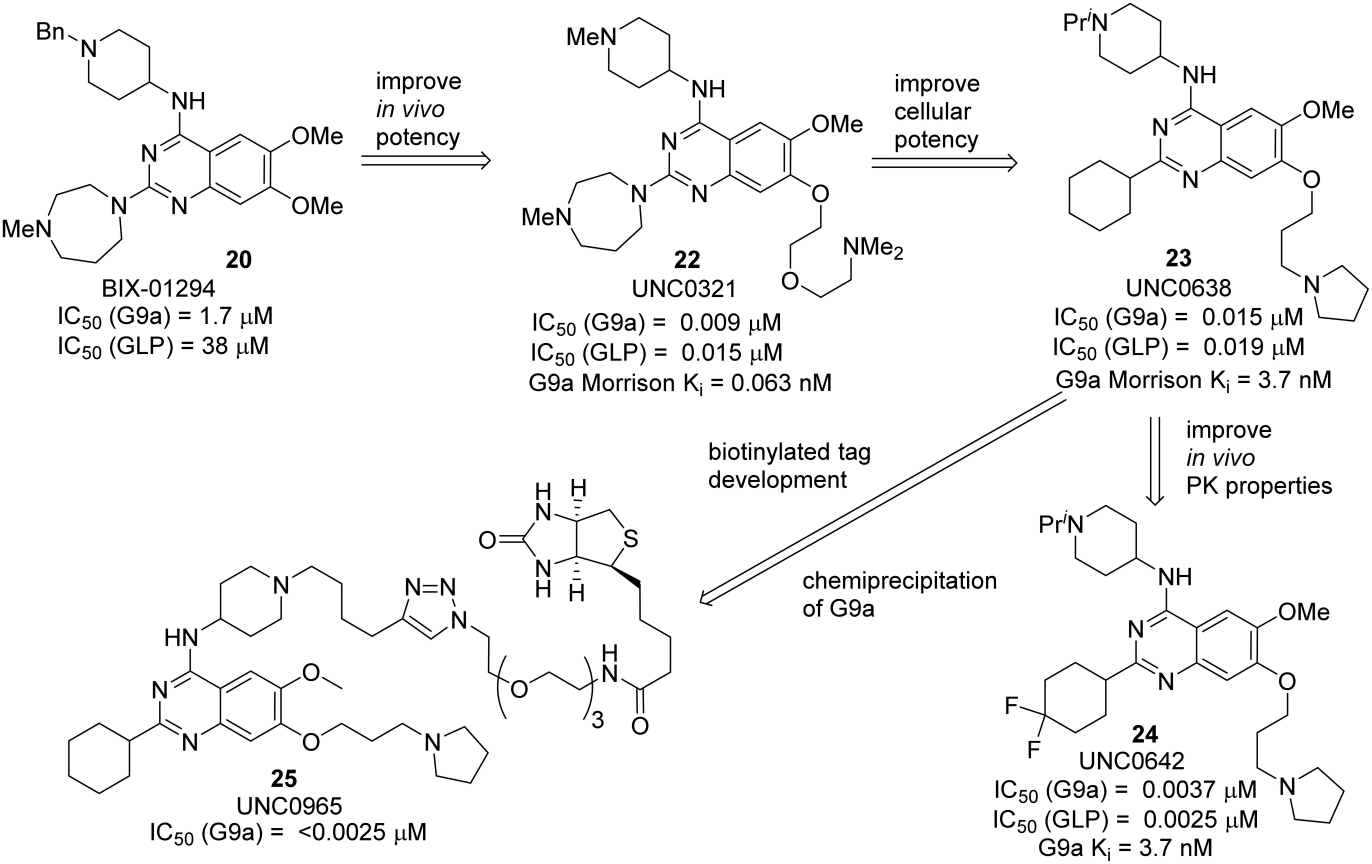
**Development of G9a-GLP inhibitors by Jin and co-workers**.

BIX 01294 **20**, the first G9a/GLP inhibitor, was discovered by Kubicek et al. (2007) by High-Throughput Screening of Compound Library against G9a (Kubicek et al., 2007) (Figure 3). The crystal structure of the catalytic SET domain of GLP in complex with BIX-01294 and S-adenosyl-L-homocysteine revealed that, the inhibitor is bound in the substrate peptide groove at the location where the histone H3 residues N-terminal to the target lysine lie in the previously solved structure of the complex with histone peptide (Chang et al., 2009). BIX-01294 **(20)** exhibited cellular toxicity at high concentration, which limited its further development. Synthesis of BIX-01294 was reported by Liu et al. (2009). In order to obtain more potent and selective inhibitors toward G9a/GLP, modification based on the structure of BIX01294 **(20)** has been carried out by Jin and co-workers since 2009 (Liu et al., 2010, 2011, 2013; Vedadi et al., 2011; Konze et al., 2014) (Scheme 2). Initial investigation on G9a-BIX01294 complex and SAR studies led to the discovery of the first selective G9a inhibitor, UNC0224 **(21)**. The corresponding X-ray crystal structure of the G9a-**21** complex, was also obtained by the same research group (Liu et al., 2009). Further crystal-structure-based optimization process resulted in the discovery of UNC0321 **(22)**, which demonstrated higher cellular potency (Morrison *K*_i_ = 63 pM) (Liu et al., 2010). Successive development based on structural design and chemical synthesis gave rise to a potent and selective inhibitor UNC0638 **(23)**, which also served as an effective chemical probe for G9a/GLP (Liu et al., 2011; Vedadi et al., 2011). Unfortunately, the poor pharmacokinetic properties of UNC0638 limited the further animal studies of this lead compound. In order to improve the *in vivo* pharmacokinetic properties of UNC0638, further optimization led to the development of a more promising lead compound UNC0642 **(24)** (Liu et al., 2013). This inhibitor displayed IC_50_ < 2.5 nM and excellent selectivity toward G9a protein over other methyl transferases. In addition, it also showed an improved *in vivo* pharmacokinetic properties. In 2014, a biotinylated tag of UNC0965 **(25)** was developed by the same research group for “chemiprecipitation” of G9a protein from whole cell lysates (Konze et al., 2014).

In 2010, Chang et al. reported a potent and less toxic G9a inhibitor, E72 **(26)** (Chang et al., 2010) (Figure 2). However, its cellular potency is lower than that of BIX-01294 **(20)**. Moreover, Yuan et al. and Sweis et al. independently reported BRD9539 **(27)** (Yuan et al., 2012) and A366 **(28)** (Sweis et al., 2014) as potent and selective G9a inhibitors in 2012 and 2014, respectively. In 2014, Srimongkolpithak et al. disclosed structures and the corresponding synthesis of potent G9a inhibitors Sirmongkolithak cmp 41 **(29)** and Sirmongkolithak cmp 41 **(30)** (Srimongkolpithak et al., 2014).

**Scheme 3:**
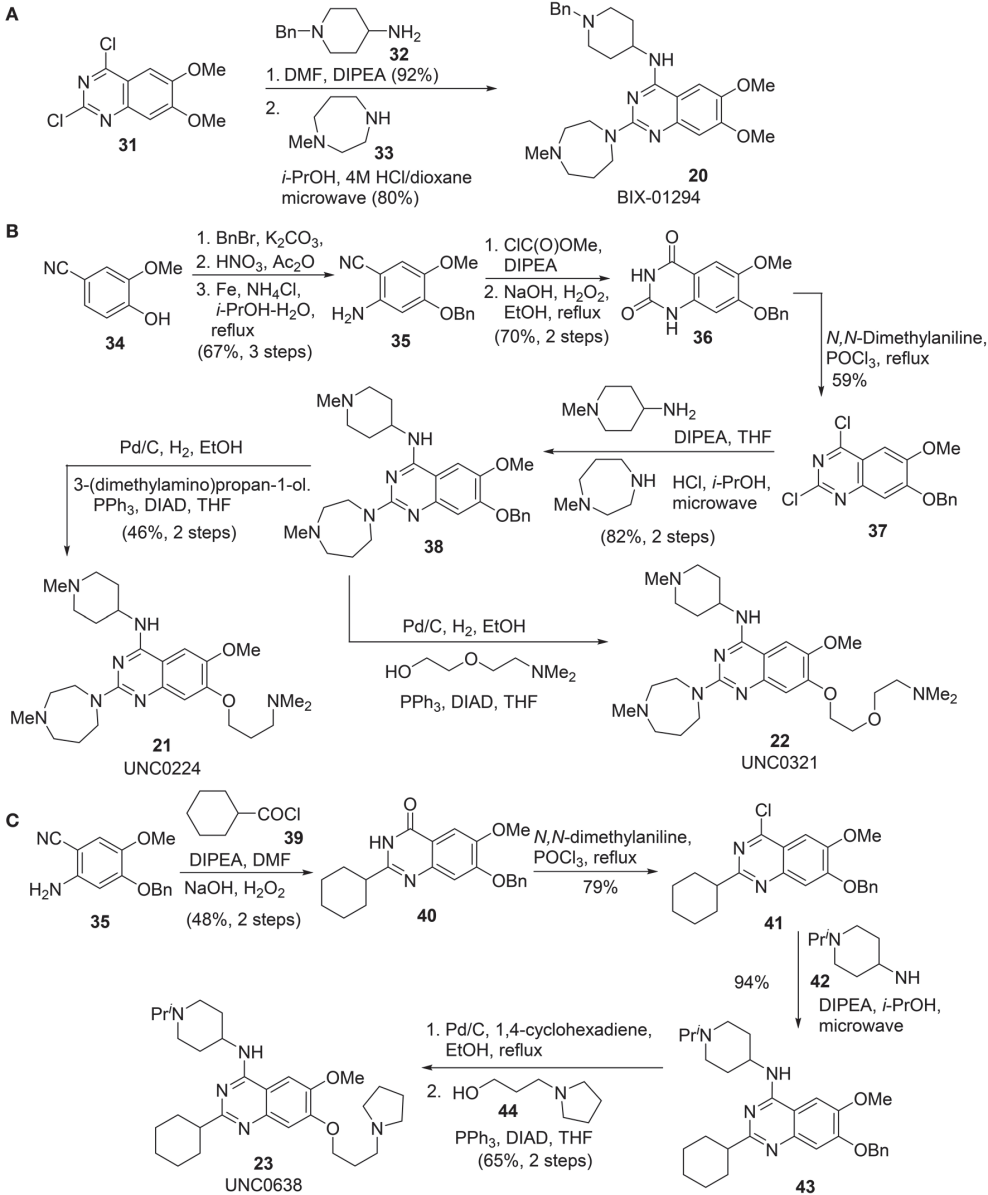
**(A)** Synthesis of BIX-01294 by Liu et al. **(B)** Synthesis of UNC0224 and UNC0321 by Liu et al. **(C)** Synthesis of UNC0638 by Vedadi et al.

Synthesis of BIX-01294 **(20)** was reported by Liu et al. (2009) (Scheme 3A). Commercially available quinazoline derivative **31** was exposed to amine **32** in the presence of Hünig's base in DMF afforded the corresponding condensation intermediate, which was then subsequently reacted with amine **33** under acidic condition in microwave to furnish BIX01294 **20** as the desired product.

Syntheses of UNC0224 **21** (Liu et al., 2009) and UNC0321 **22** (Liu et al., 2010) were reported by Liu et al. (2009, 2010), respectively. (Scheme 3B) Successive benzylation, nitration, and reduction of commercially available **34** afforded aniline **35** in good yield. Treatment of aniline **35** with methyl chloroformate followed by hydrolysis of the nitrile group and simultaneous ring closure produced quinazolinedione **36** in 70% yield over two steps. Dichloroquinazoline **37**, obtained by the treatment of **36** with POCl_3,_ was subjected to two sequential chlorine displacement reactions with two different amines to afford **38** in 82% yield over two steps. With the key intermediate **38** in hand, the synthesis of UNC0224 **21** and UNC0321 **22** was completed by reductive debenzylation followed by elongation of the resultant phenol under Mitsunobu condition.

Synthesis of UNC0638 **(23)** was reported by Vedadi et al. (2011) (Scheme 3C). Intermediate **35**, which has been employed in the synthesis of UNC0224 **(21)** (Liu et al., 2009), can be selected as the starting material for the construction of UNC0638 **(23)**. Acylation of **35** with cyclohexanoyl chloride **39** followed by an oxidative cyclization afforded **40** in 48% yield over two steps. Chloroquinazoline **41**, obtained by the treatment of **40** with POCl_3_, was then condensed with amine **42** to give rise to **43**. Successive debenzylation followed by elongation of the resultant phenol under Mitsunobu condition produced UNC638 **(23)** in 65% yield.

**Scheme 4:**
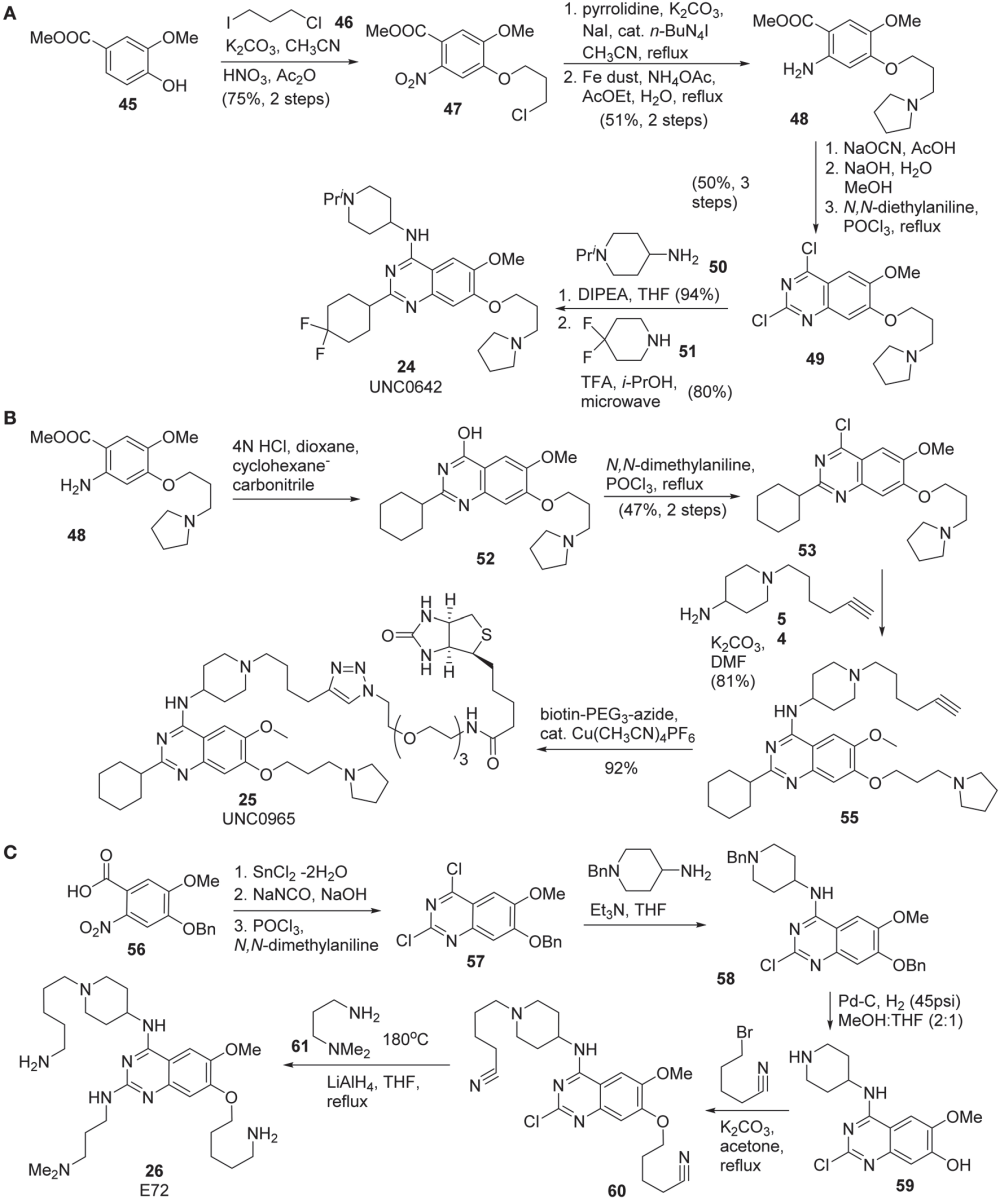
**(A)** Synthesis of UNC0642 by Liu et al. **(B)** Synthesis of UNC0965 by Konze et al. **(C)** Synthesis of E72 by Chang et al.

Synthesis of UNC0642 **(24)** was reported by Liu et al. (2013) (Scheme 4A). Commercially available benzoate **45** was subjected to nucleophilic substitution with 1-chloro-3-iodopropane **46** followed by nitration gave rise to **47** in 75% yield over two steps. Substitution of chlorine of **47** with pyrrolidine and reduction of nitrate with Fe dust afforded **48**. Dichloro-quinazoline **49** was prepared from this intermediate in 50% yield via a three-step sequence including urea formation, cyclization followed by chlorination. The consecutive displacement of two chlorine atoms with two different amines produced UNC0642 **(24)** in 75% yield.

Synthesis of UNC0965 **25** was reported by Konze et al. (2014) (Scheme 4B). Cyclization of the known intermediate **48** afforded **52** and subsequent chlorination furnished **53** in 47% yield over two steps. Treatment of this intermediate with amine **54** afforded **55**, which was then subjected to a Copper-catalyzed click reaction with commercially available biotin-PEG_3_-azide to afford UNC0965 **(25)** in 92% yield.

The preparation of E72 **(26)** was disclosed by Chang et al. (2010) (Scheme 4C). This synthesis is straight-forward, however, no yield for each step of the synthesis was reported. The starting material **56**, prepared according to the previously described procedure (Thurston et al., 1990), was reduced by the action of SnCl_2_ (Hu et al., 2001) and then converted into dichloroquinazoline **57** via a two step sequence including treatment of the acid with sodium isocyanate followed by chlorination by the use of POCl_3_(Andrus et al., 2002; Smits et al., 2008). Displacement of the more reactive chlorine atom with 4-amino-1-benzylpiperidine afforded **58**, which was subjected to catalytic hydrogenolysis to furnish **59**. Treatment of **59** with 5-bromopentanenitrile in the presence of K_2_CO_3_ in refluxing acetone to produce **60**. Displacement of chlorine atom with N, N, -dimethylaminopropylamine at high temperature followed by the reduction of nitriles to primary amines using lithium aluminum hydride to furnish E72 **(26)** as the desired product.

**Scheme 5:**
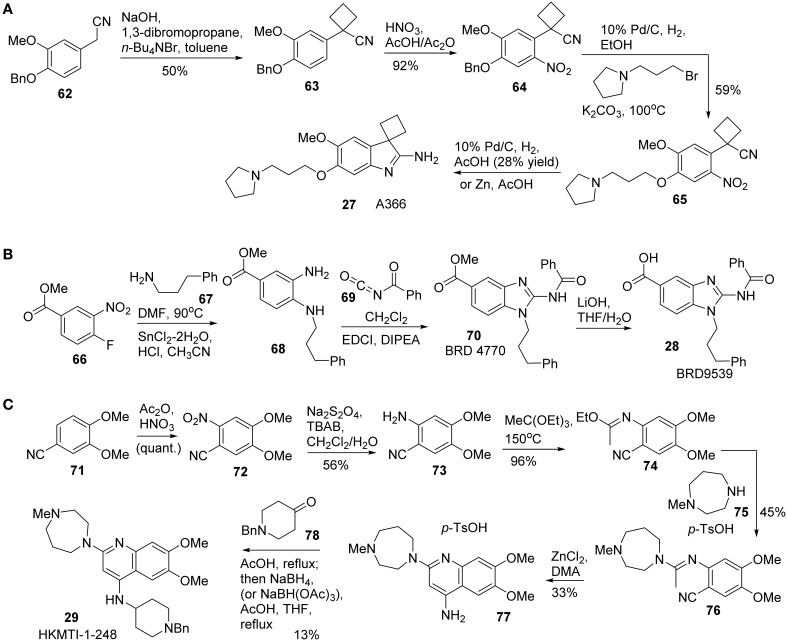
**(A)** Synthesis of A366 by Sweis et al. **(B)** Synthesis of BRD9539 by Yuen et al. **(C)** Synthesis of HKMTI-1-248 by Srimongkolpithak et al.

Synthesis of A366 **(27)** was reported by Sweis et al. (2014) (Scheme 5A). Treatment of **62** with 1,3-dibromopropane under basic condition afforded the corresponding cyclobutane-containing derivative **63**, which was then subjected to nitration to give rise to **64**. Successive debenzylation and nucleophilic substitution with 1-(3-bromopropyl)pyrrolidine produced **65**. Intramolecular cyclization was accomplished under reductive conditions to furnish A366 **(27)** in 28% yield.

Synthesis of BRD9539 **(28)** was disclosed by Yuan et al. (2012). The detailed synthesis is shown in Scheme 5B, however, no yield was reported for the synthesis. Nucleophilic aromatic substitution of fluorobenzoate derivative **66** with phenylpropylamine **67** followed by a reduction of nitro group with SnCl_2_ afforded **68**. Treatment of **68** with benzoyl isocyanate **69** afforded the corresponding benzoyl urea, which was then undergoing a EDCI-mediated cyclization to give rise to BRD4770 **(70)**. Saponification of **70** produced BRD9539 **(27)**.

Quinoline derivative HKMTI-1-248 was reported by Srimongkolpithak et al. (2014). The synthesis commenced from the nitration of commercially available substrate **71** afforded **72**. A sequential reduction of **72** to the corresponding amine **73**, followed by condensation of the resulting amine with triethyl orthoacetate at high temperature to afford *N*-arylimidic ester **74**. Treatment of **74** with 1-methylhomopiperazine **75** in the presence of *p*-toluenesulfonic acid produced **76**, which was then subjected to a ZnCl_2_-promoted ring closure process to afford **77** (Moore and Kornreich, 1963). Condensation of aniline **77** with ketone **78** followed by a reductive amination to furnish HKMTI-1-248 (Scheme 5C).

## EZH2

EZH2 is one of the first histone lysine methyltransferases found to be related to human cancers (Varambally et al., 2002; Kleer et al., 2003). EZH2 over expression has been linked to breast, prostate, and bladder cancers. EZH2/EZH1 is one of the core components within the PRC2 complex while EZH2 is the enzymatic subunit responsible for histone H3K27 methylation (Czermin et al., 2002; Kuzmichev et al., 2002; Muller et al., 2002; Margueron and Reinberg, 2011). It has been reported that ablation of EZH2 in tumor cells using RNA interference technology suggested that the enzyme is a promising drug target for cancer treatment (Varambally et al., 2002).

**Figure 4:**
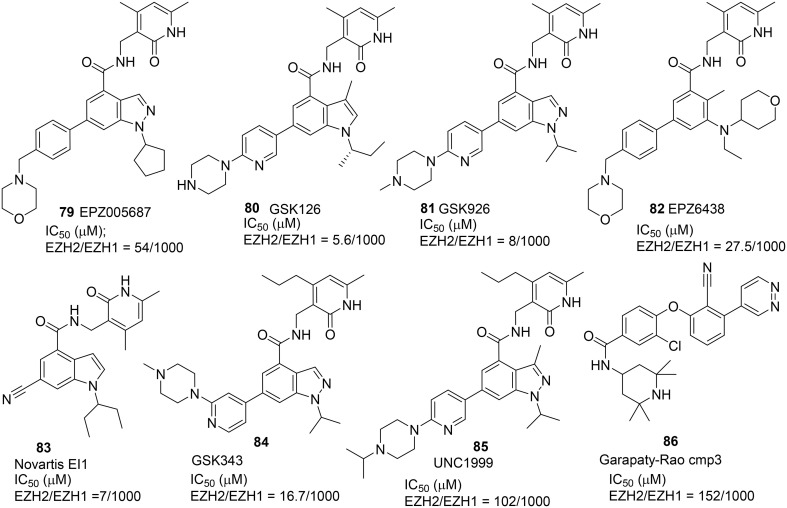
**Example of inhibitors targeting protein EZH**.

Three selective and potent EZH2 inhibitors namely EPZ005687 **(79)** (Knutson et al., 2012), GSK126 **(80)** (Mccabe et al., 2012), and Novartis EI1 **(81)** (Qi et al., 2012) were reported in 2012 (Figure 4). These inhibitors exhibited high selectivity over other histone methyltransferase. Verma et al. disclosed a high potent EZH2 inhibitor GSK343 **(84)** (Verma et al., 2012), while Konze et al. reported a selective EZH2 inhibitor UNC1999 **(85)** which possesses structural characteristics similar to those of EPZ005687 **(79)** and GSK 126 **(80)** with a pyridine-amide scaffold (Konze et al., 2013). UNC1999 **(85)** has better physicochemical properties and oral bioavailability when compared with previously reported inhibitors. Further optimization led to the development of the latest version of EZH2 inhibitor EPZ-6438 **(82)**, which exhibited excellent potency, selectivity, and pharmacokinetic properties over EPZ005687 **(79)** (Knutson et al., 2013, 2014), and which has recently progressed into Phase-I trial for treatment of lymphoma.

**Scheme 6:**
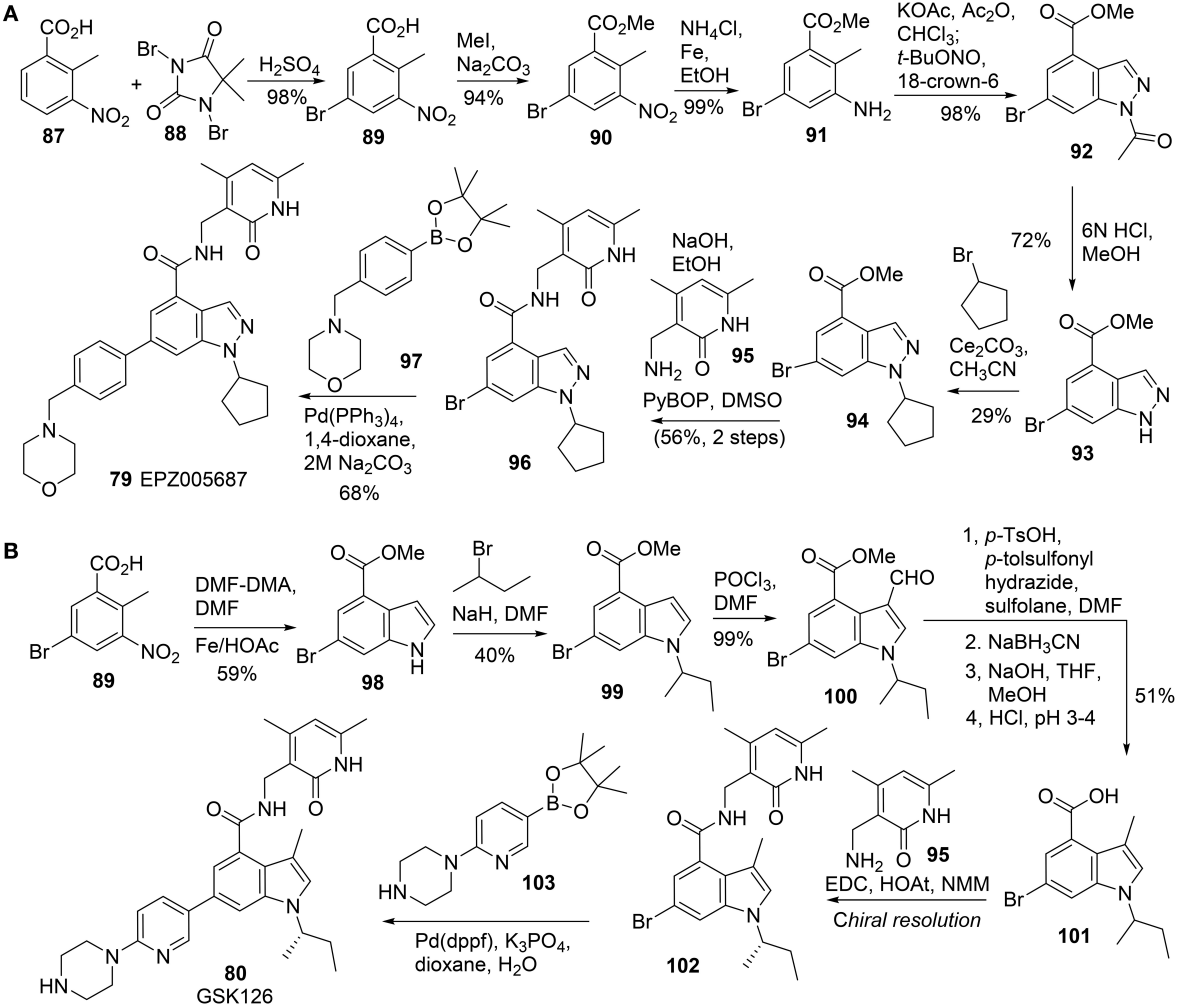
**(A)** Synthesis of EPZ005687 by Kuntson et al. **(B)** Synthesis of GSK126 by McCabe et al.

All these EZH2 inhibitors (Figure 4) are SAM-competitive. So far, no synthesis for Novartis EI1 **(83)** has been reported. The synthesis of EPZ-6438 **(82)** was described in a patent (WO2012142504). Synthesis of EPZ005687 **(79)** was reported by Knutson et al. (2012) (Scheme 6A). *meta*-Selective bromination of benzoic acid derivative **87** with 1,3-dibromo-5,5-dimethyl-2,4-imidzaolidinedione (DBDMH) **88** afforded **89** in 98% yield (Eguchi et al., 1994). Methylation of benzoic acid group of **89** using sodium carbonate and iodomethane afforded ester **90**, which was then converted into aniline **91** by reduction of the nitro group. Acetylation of aniline **91** followed by nitrozation with *tert*-butyl nitrite under phase-transfer condition afforded indazole derivative **92**, which was subjected to deacetylation to afford **93** in 72% yield. Alkylation of **93** with bromocyclopentane was facilitated by cesium carbonate to give rise to **94** in 29% yield. Saponification of **94** afforded the free carboxylic acid, which was condensed with amine **95** by the action of PyBop to produce **96** in 56% yield. Suzuki coupling between aryl bromide **96** and boronate **97** furnished EPZ005687 **(79)** in 68% yield.

GSK126 **(80)** was prepared by Mccabe et al. (2012) (Scheme 6B), according to an approach which is similar to the one reported by Knutson (Knutson et al., 2012). Following Leimgruber-Batcho indole synthesis, methyl 5-bromo-2-methyl-3-nitrobenzoate **89** was readily converted into methyl 6-bromo-1H-indole-4-carboxylate **98** in 59% yield. (Batcho and Leimgruber, 1985) Alkylation of indole nitrogen of **98** with 2-bromobutane followed by formylation of **99** at C-3 position delivered **100** in 40% yield over two steps. Deoxygenative reduction of aldehyde **100** was mediated by *N*-tosylhydrazine with sodium cyanoborohydride as the reductant to afford the corresponding reduction product, which was then saponified to the corresponding acid **101**. Condensation of acid **101** with amine **95** was mediated by EDC-HOAt to furnish the corresponding amide which was then subjected to chiral resolution to afford the desired (*S*)-isomer **(102)**. Suzuki cross-coupling of aryl bromide A **102** and boronate **103** completed the synthesis of GSK126 **(80)**.

**Scheme 7:**
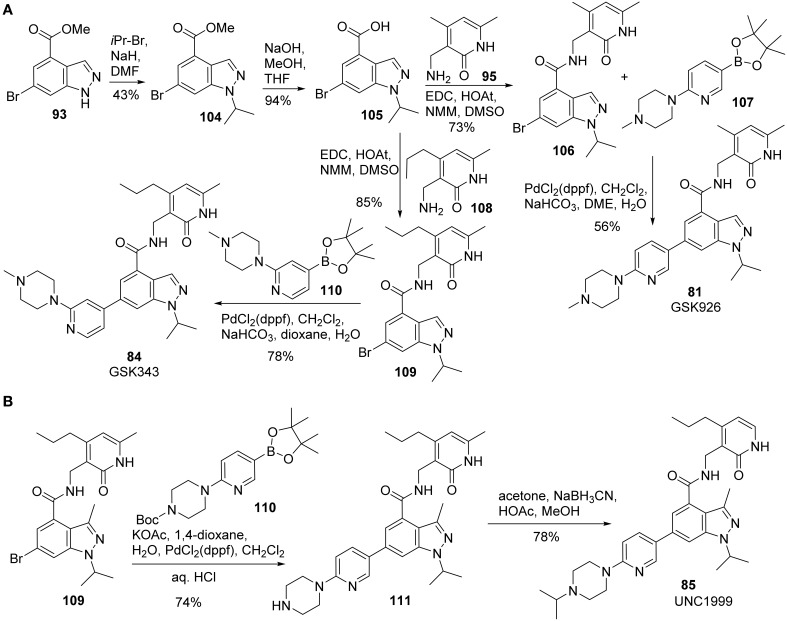
**(A)** Synthesis of GSK926 and GSK343 by Verma et al. **(B)** Synthesis of UNC1999 by Konze et al.

Synthesis of GSK926 **(81)** and GSK 343 **(84)** was reported by Verma et al. (2012) (Scheme 7A). Alkylation of **93** with 2-bromoisopropane afforded **104** in 43% yield. Saponification of **104** followed by coupling of the resultant acid **105** with amine **95** furnished amide **106**, which was then subjected to Suzuki coupling with boronic ester **107** to give rise to GSK926 **(81)** in 56% yield. GSK343 **(84)** was prepared following the sequences described for the synthesis of GSK926 **(81)**. (Scheme 7A) Thus, condensation of acid **105** and amine **108** produced the corresponding amide **109**, which underwent a highly efficient Suzuki coupling reaction with **110** to complete the synthesis.

Synthesis of UNC1999 **(85)** was reported by Konze et al. (2013) (Scheme 7B). The synthetic strategy for UNC1999 **(85)** is similar to that employed for the preparation of GSK926 **(81)** and GSK343 **(84)** (Verma et al., 2012). With the key intermediate **109** in hand, Suzuki coupling of **109** with boronate **110** followed by deprotection of Boc group afforded **111** in 74% yield. Reductive amination of the second amine with acetone by sodium cyanoborohydride then furnished UNC1999 **(85)** in 85% yield.

**Scheme 8:**
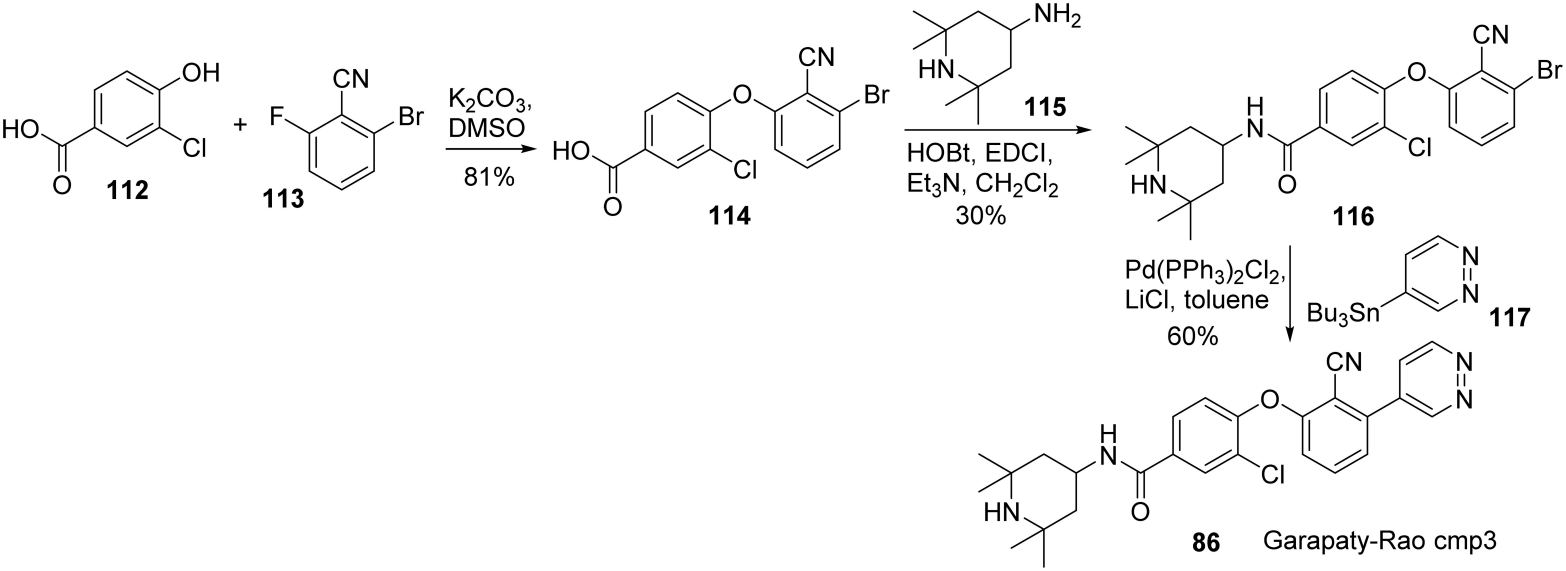
**Synthesis of Garapaty-Rao cmp3 by Nasveschuk et al**.

Synthesis of Garapaty-Rao cmp3 **(86)** (Garapaty-Rao et al., 2013) was accomplished by Nasveschuk et al. and disclosed in 2014 (Nasveschuk et al., 2014) (Scheme 8). Treatment of phenol **112** with fluoroarene **113** underwent a S_N_Ar reaction to afford biaryl ether **114** in 81% yield (Zhu, 1997). Condensation of acid **114** with amine **115** was facilitated by the action of EDCI to furnish amide **116**. Stille coupling of freshly prepared **116** with tributyltin derivative **117** under standard condition afforded cmp3 **(86)** in 60% yield.

## DOT1L

DOT1 and DOT1L (DOT1-Like protein) are the only known enzymes which targeted H3K79 on the nucleosome core (Feng et al., 2002; Lacoste et al., 2002; Ng et al., 2002; Van Leeuwen et al., 2002), and it methylates on the lysine residue (Frederiks et al., 2008). DOT1L was later found to be important for telomeric silencing. DOT1L-mediated H3K79 methylation was found to be related to transcriptional activation, which involves regulation of a wide range of biological processes, including transcriptional regulation, DNA repair, cell cycle regulation and myocardial genesis (Nguyen and Zhang, 2011). Unlike other histone methyltransfer, Dot1 does not contain a SET domain, and it specifically methylates nucleosomal histone H3 (Min et al., 2003). Moreover, DOT1L also interacts with interact with MLL (mixed lineage leukemia) fusion proteins (Ayton and Cleary, 2001; Okada et al., 2005; Nguyen and Zhang, 2011), which would result in the misregulation of hox gene expression, and lead to lukemogensis. Significant advances in the development of DOT1 inhibitors for acute leukemias bearing MLL rearrangements have led to the first reported histone methyltransferase inhibitor to enter human clinical trials (Daigle et al., 2013).

**Figure 5:**
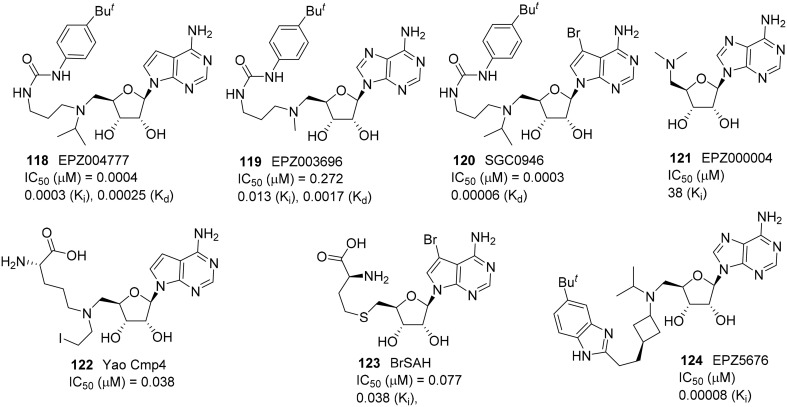
**Example of inhibitors targeting protein DOL1L**.

A small molecule SAM-mimic compound called EPZ004777 **(118)** was identified as a potent DOT1L inhibitor by Daigle et al. (2011) (Figure 5). It selectively inhibited H3K27 methylation with high potency (IC_50_ = 0.4 nM) and suppress lukemogenesis by selectively killing leukemic cells that bear MLL translocation genes (Daigle et al., 2011). Later, the same research group reported the DOT1L inhibitor EPZ003696 **(119)** is 43 times less potent than EPZ004777 **(118)** on DOT1L inhibition (Basavapathruni et al., 2012). Meanwhile, Yu et al. reported the brominated analog of EPZ004777, SGC946 **(120)**, exhibited more significant potency in cell than that of EPZ004777 **(118)** (Yu et al., 2012). In 2013, Daigle et al. identified the optimized SAM-mimic, EPZ5676 **(124)**, is a potent DOT1L inhibitor that causes tumor regressions in a rat xenograft model of MLL-rearranged leukemia. This inhibitor was more than 37 000-fold selective for DOT1L over 16 other protein methyltransferases and has entered a Phase I clinical trial (Daigle et al., 2013). Studies on pharmokinetics and metabolism of EPZ5676 **(124)** were also reported (Basavapathruni et al., 2014). EPZ5676 **(124)** was developed by Epizyme and the detailed synthesis was reported in a patent (Olhava et al., 2012). Besides, Yao et al. and Yu et al. revealed two highly potent and selective DOT1L inhibitors, Yao cmp4 **(122)** (Yao et al., 2011, 2012) and BrSAH **(123)** (Yu et al., 2013b), in 2011 and 2013, respectively. Yao cmp4 **(122)** inhibits DOT1L as a SAM analog at the SAM binding site. Discovery of BrSAH **(123)** by Yu et al. demonstrated that the addition of a single halogen at the critical position of cofactor SAH (by-product of SAM DOT1L-catalyzed histone methylation) could result in a 8-fold increase in potency against DOT1L. All these DOT1L inhibitors (Figure 5) shown are SAM-competitive.

**Scheme 9:**
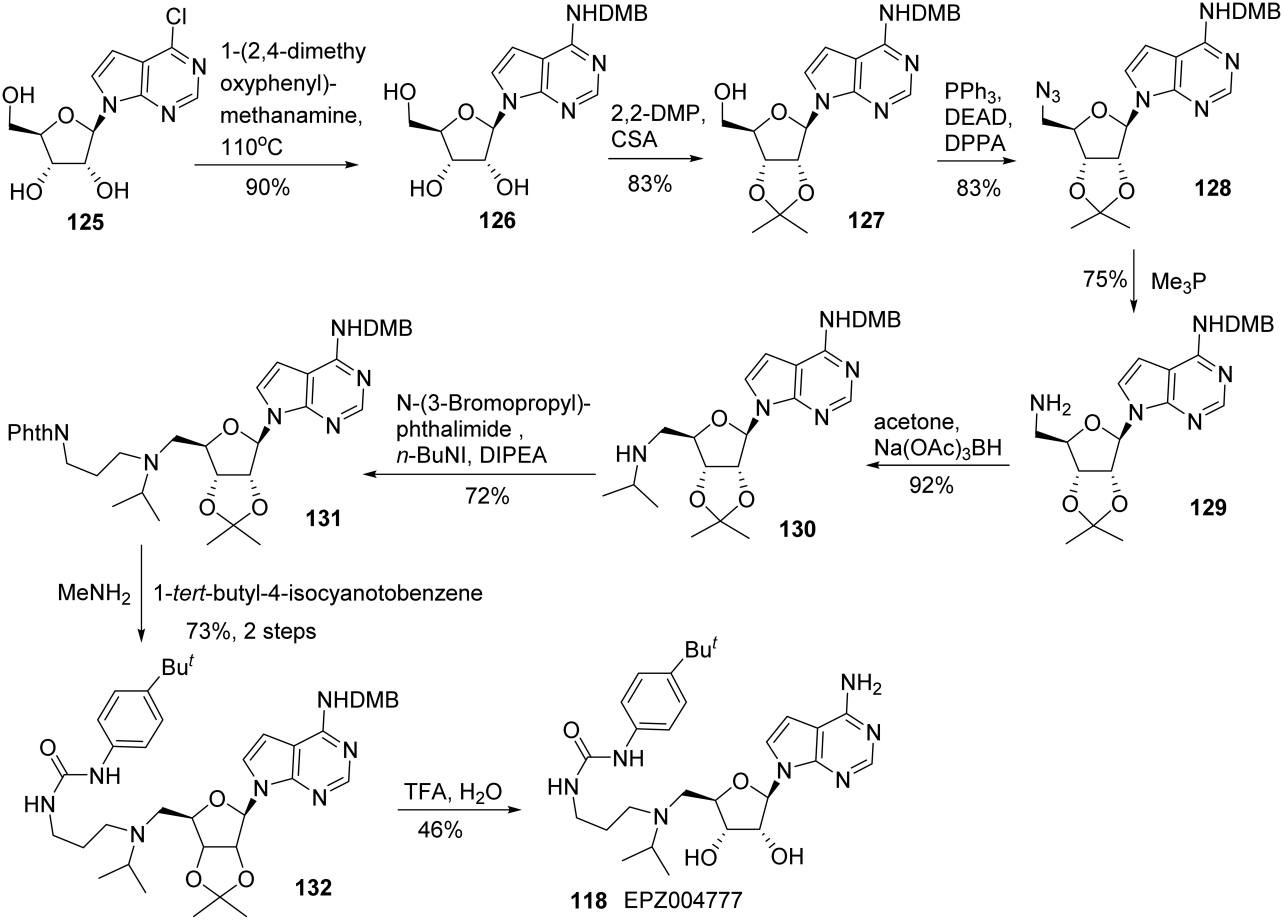
**Synthesis of EPZ004777 by Basavapathruni et al**.

Synthesis of EPZ004777 **(118)** was reported by Basavapathruni et al. (2012) (Scheme 9). Treatment of 7-chloro tubercidin **(125)** with 1-(2,4-dimethyoxyphenyl) methanamine at 110°C afforded **126** in 90% yield. The *cis*-diol of **126** was protected as its acetonide **127** and the remaining primary alcohol was then converted into its corresponding azide **128** under Mitsunobu condition. The azide moiety of **128** was reduced to amine **129** by the action of PMe_3_, followed by a reductive amination to give rise to secondary amine **130**. *N*-alkylation of amine **130** with *N*-(3-Bromopropyl)phthalimide furnished **131** in 72% yield. Cleavage of the *N*-phthaloyl group of **131**, followed by coupling of the resultant primary amine with 1-*tert*-butyl-4-isocyanotobenzene to afford the desired urea derivative **132** in 73% yield over two steps. Finally, deprotection of both acetonide and DMP protecting groups using TFA produced EPZ004777 **(118)** in 46% yield.

**Scheme 10:**
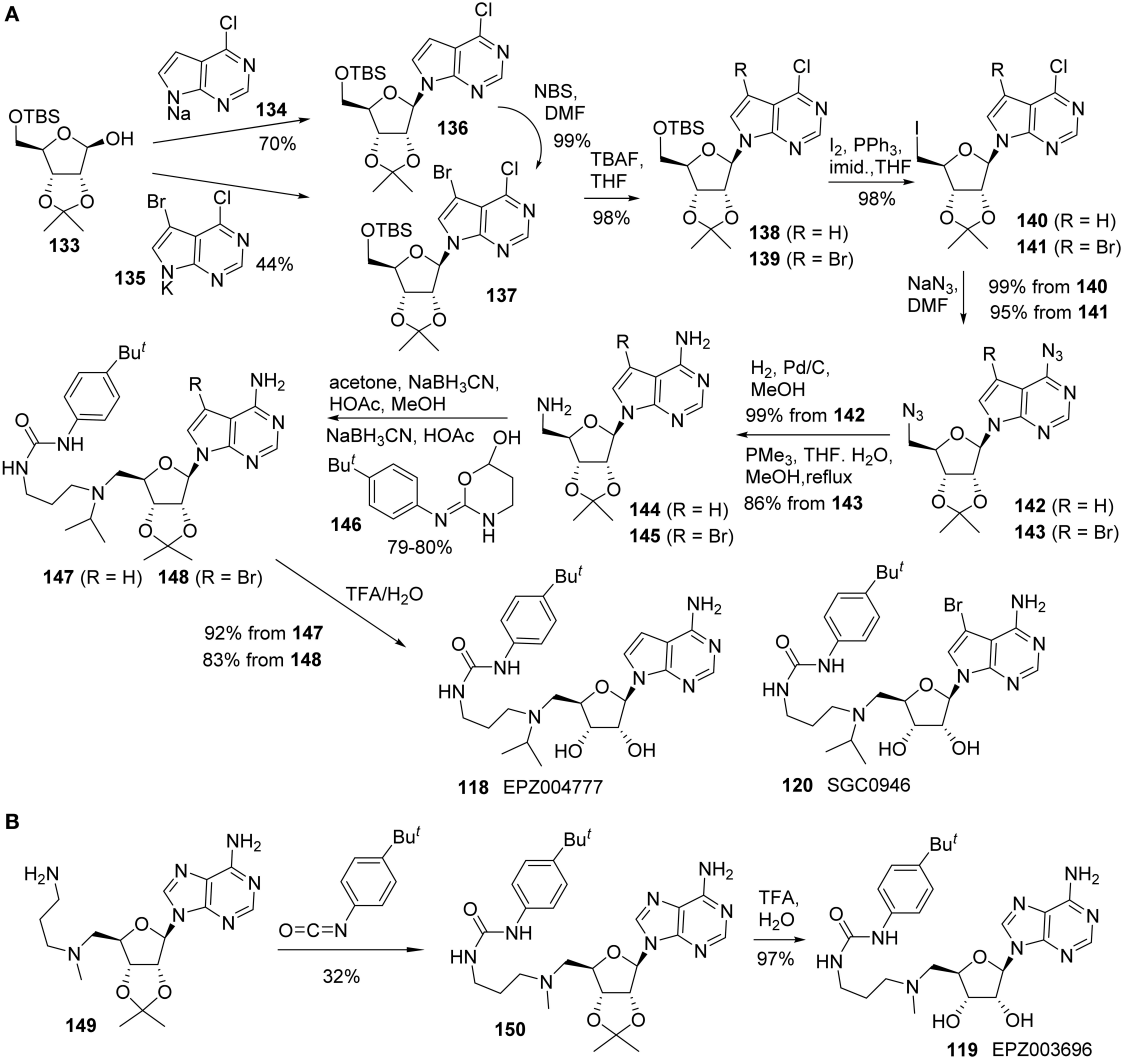
**(A)** Synthesis of EPZ004777 and SGC0946 by Yu et al. **(B)** Synthesis of EPZ003696 by Basavapathruni et al.

Yu and co-workers reported the synthesis of EPZ004777 **(118)** using a different route in which both the primary amine and pyrimidin-4-amine were installed via the corresponding azide substitution and subsequent reduction (Yu et al., 2012, 2013a). In addition, a brominated analog of EPZ004777 **(118)**, named SGC946 **(120)**, was also synthesized by the same research group (Yu et al., 2012, 2013a) (Scheme 10A). Thus, treatment of *bis*-protected D-ribose **(133)** with **134** in the form of sodium salt afforded **136** in 70% yield. Similar reaction with **135** afforded **137** in 44% yield. **137** could also be obtained by bromination of **136** in essentially quantitative yield. Amine **144** could be constructed from the key intermediate **136** by sequential transformations including cleavage of the TBS protecting group, conversion of the resultant primary alcohol into the corresponding iodide, azide displacement followed by catalytic hydrogenation of the resultant azide derivative. Amine **144** was subjected to two sequential reductive amination processes to afford urea derivative **147** in 80% yield over two steps. Both acetone and oxazinanol **146** were employed as reactants during the respective reductive amination process (Scheme 10A). Acid hydrolysis of the acetonide protecting group furnished EPZ004777 **(118)** in 92% yield.

Amine **145** was converted into SGC0946 **(120)** using the same sequence of reactions and Staudinger reduction was employed for the conversion of azide **143** to the corresponding amine **145**.

Synthesis of EPZ003696 **(119)** was reported by Basavapathruni et al. (2012) (Scheme 10B). The synthesis began with the known amine **149**, which was prepared as previously described (Mccloskey et al., 2009). Treatment of amine **149** with 1-*tert*-butyl-4-isocyanotobenzene afforded the corresponding urea derivative **150** in 32% yield. TFA-mediated removal of acetonide afforded EPZ003696 **119** in 97% yield.

**Scheme 11:**
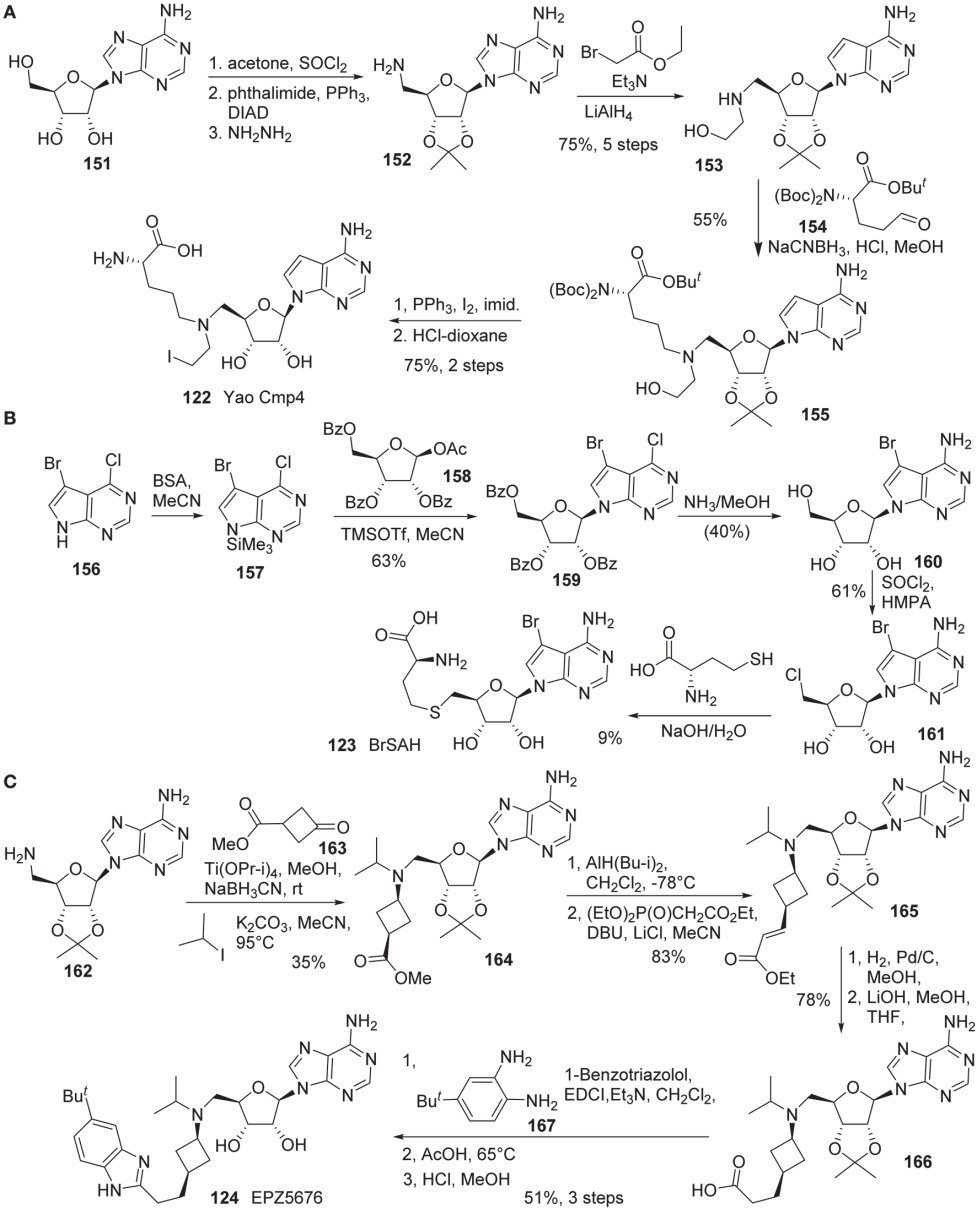
**(A)** Synthesis of Yao cmp4 by Yao by Yu et al. **(B)** Synthesis of BrSHA by Yu et al. **(C)** Synthesis of EPZ5676 by Olhava et al.

Synthesis of Yao cmp4 **(122)** is illustrated in Scheme 11A (Yao et al., 2011). The *cis*-diol of adenosine **151** was protected as the corresponding acetonide. Direct *N*-alkylation of a phthalimide with the primary alcohol under Mitsunobu conditions afforded the corresponding *N*-phthaloyl protected amine, which was cleaved with hydrazine to give rise to amine **152**. This amine **(152)** was alkylated with ethyl bromoacetate followed by reduction of the ethyl ester with LiAlH_4_ to afford **153** in 75% yield over five steps. Reductive amination of **153** with aldehyde **154** furnished **155** in 55% yield. Conversion of the hydroxy group to the corresponding iodide was mediated by the action of PPh_3_, iodine and imidazole. Global deprotection of the resultant iodide furnished Yao Cmp4 **(122)** in 75% yield over two steps.

Synthesis of BrSAH **(123)** was reported by Yu et al. (2013b) (Scheme 11B). Treatment of 5-bromo-4-chloro-7H-pyrrolo[2,3-d]pyrimidine **160** with *N*,*O*-Bis(trimethylsilyl)-acetamide (BSA) in acetonitrile furnished silylated intermediate **157**, which was subjected to glycosylation with **158** to afford **159** in 63% yield (Seela and Ming, 2007). Simultaneous displacement of chlorine atom with ammonia and hydrolysis of benzoyl groups furnished **160** in 40% yield. The primary hydroxy group of **160** was converted into the corresponding chloride with SOCl_2_to give rise to **161** in 61% yield. Nucleophilic substitution of **161** with L-homocysteine under alkaline conditions afforded the desired product BrSAH **123** in 9% yield.

The detailed synthesis of EPZ5676 **(124)** was described by Epizyme in a patent (Olhava et al., 2012) (Scheme 11C). Thus, 5′-Amino-5′-deoxy-2′,3′-O-(1-methylethylidene)-adenosine **(162)** was transformed into the corresponding bis-alkylated product **(164)** in a two step sequence involving reductive amination with ketone **163**, and alkylation with isopropyl iodide. Dibal reduction of **164** in dichloromethane at -78°C provided the corresponding aldehyde, which underwent Wadsworth-Emmons olefination to afford **165** in 83% yield in two steps. Catalytic hydrogenation of α,β-unsaturated ester, followed by saponification of the ethyl ester by treatment with aqueous lithium hydroxide in methanl/tetrahydrofuran afforded acid **166** in 78% yield. Acid **166** was converted into EPZ5676 **(124)** in 51% yield by a three-step sequence involving EDCI-promoted coupling of acid **166** with diamine **167**, acid-mediated benzimidazole cyclization and cleavage of the acetonide moiety.

## SET8

SET8 (also known as PR-Set7/9, SETD8, KMT5A), a member of the SET domain-containing methyltransferase family is solely responsible for the catalysis of monomethylation of histone protein H4K20 (Fang et al., 2002; Nishioka et al., 2002; Beck et al., 2012). Since H4K20 methylation is widespread in the genome and appears to be regulated during the cell cycle, SET8 plays important roles in various of biological processes, which involved in regulation of a range of biological processes, namely gene transcription (Congdon et al., 2010; Li et al., 2011), cell-cycle progression and development (Jorgensen et al., 2007; Abbas et al., 2010; Centore et al., 2010; Wu et al., 2010), genome integrity (Houston et al., 2008; Oda et al., 2009), DNA replication, DNA double-strand breaks and DNA damage response (Dulev et al., 2014). Moreover, SET8 demonstrated its ability on methylation of non-histone proteins including p53 tumor suppressor protein (Shi et al., 2007). This methylation process resulted in the suppression of p53 mediated transcription activation of highly responsive target genes. In addition, SET8 catalyzes monomethylation of proliferating cell nuclear antigen (PCNA) that results stabilization of PCNA protein and consequentially promotes cancer cell proliferation (Takawa et al., 2012). SET8 also played a novel role in tumor invasion and metastasis and provided a molecular mechanism underlying TWIST-promoted EMT (Yang et al., 2012).

**Figure 6:**
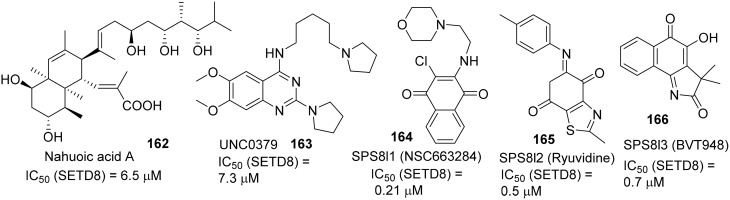
**Example of inhibitors targeting protein SETD8**.

**Scheme 12:**
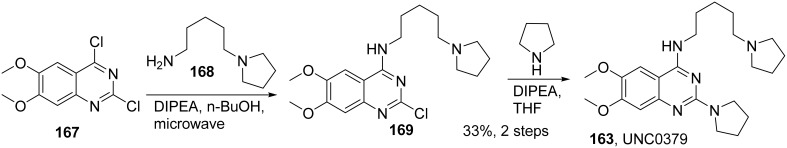
**Synthesis of UNC0379 by Ma et al**.

Selective small-molecule inhibitors of SET8 are important tools for investigating the biology of this emerging target. However, only a limited number of selective inhibitors of SET8 have been reported (Williams et al., 2013; Blum et al., 2014; Ma et al., 2014a,b) (Figure 6). Marine natural product nahuoic acid A **(162)**, the first known selective inhibitor of SET8, is competitive with the cofactor SAM and non-competitive with the peptide substrate (Williams et al., 2013). Very recently, Ma et. al. disclosed the structure of UNC0379 **(163)**, which is the first synthetic small molecule inhibitor of SET8 (Ma et al., 2014a,b). Later, Blum et al. reported the validation of three irreversible SET8 methyltransferase inhibitors with different mode of inhibition. (Blum et al., 2014). Biological studies demonstrated that the inhibition of SET8 by SPS8I1 **(164)** and SPS8I3 **(166)** are substrate-dependent while SPS8I2 **(165)** facilitates substrate-independent inhibition of SET8 (Blum et al., 2014). Nahouic acid A is SAM-competitive inhibitor of SET8 (Williams et al., 2013). However, no total synthesis has been reported up to date. UNC0379 is a substrate-competitive inhibitor (Ma et al., 2014a), and the corresponding synthesis is shown in Scheme 12. Treatment of commercially available 2,4-dichloro-6,7-dimethoxyquinazoline **(167)** with 5-(pyrrolidin-1-yl)pentan-1-amine **(168)** and *N*,*N*-diisopropylethylamine at room temperature afforded **169**, which was then reacted with pyrrolidine in the present of *N*,*N*-diisopropylethylamine and under microwave irradiation to give rise to UNC0379 (163) in 33% yield over two steps. (Ma et al., 2014a).

## Conclusions

Lysine methyltransferases continue to be an attractive drug design target for the treatment of various disease ranging from cancer, inflammation, psychiatric disorders to rheumatoid arthritis. This review outlines the development and chemical synthesis of a variety of structural chemotypes of lysine methyltransferase inhibitors that have been reported during the recent decade. The determination of the X-ray structure of a number of mechanism-based inhibitors and lysine methyltransferase complexes have provided important information for the structure-based design of potent lysine methyltransferase inhibitors. Many of these inhibitors exhibited impressive intrinsic potency. Subsequent lead optimization provided a broad range of lysine methyltransferase inhibitors with promising pharmacological properties. In recent years, a few of these new classes of lysine methyltransferase inhibitors have been advanced to pre-clinical development, and selective inhibitors of DOT1L and EZH2, have entered phase I clinical studies. The major issue that needs to be addressed is the selectivity among the different histone methyltransferases. By applying a non-competitive approach that preserves the physiological nature of endogenousl ligand-methyltransferase signaling, it may be possible to produce therapeutic agents that are safer than conventional competitive drugs. We predict that efforts through structure-based design would lead to the development of novel allosteric lysine methyltransferase inhibitors with superior selectivity and improving the overall side-effect profile.

### Conflict of interest statement

The authors declare that the research was conducted in the absence of any commercial or financial relationships that could be construed as a potential conflict of interest.

## Abbreviations

2,2-DMP: 2,2-dimethoxypropane
BSA: N,O-Bis(trimethylsilyl)acetoamide
DIAD: Diisopropyl azodicarboxylate
DIPEA: Diisopropylethylamine
DMAPA: dimethylaminopropylamine
DMPU: 1,3-Dimethyl-3,4,5,6-tetrahydro-2(1H)-pyrimidinone
HMPA: Hexamethylphosphoramide
HMTs: histone methyltransferases
MLL: mixed lineage leukemia
NMM: N-Methylmorpholine
PKMTs: protein lysine methyltransferases
SAH: S-Adenosyl-L-homocysteine
SAM: S-Adenosyl-L-methionine
SAR: structure-activity relationship
V-70: 2,2′-azobis-(2,4-dimethyl-4-methoxyvaleronitrile).
